# Phenethyl Isothiocyanate Exposure Promotes Oxidative Stress and Suppresses Sp1 Transcription Factor in Cancer Stem Cells

**DOI:** 10.3390/ijms20051027

**Published:** 2019-02-27

**Authors:** Bijaya Upadhyaya, Yi Liu, Moul Dey

**Affiliations:** Department of Health and Nutritional Sciences, South Dakota State University, Box 2275A, Brookings, SD 57007, USA; bupadhyaya2@unl.edu (B.U.); liu.yi1@mayo.edu (Y.L.)

**Keywords:** ALDH1, apoptosis, cancer stem cells, phenethyl isothiocyanate, reactive oxygen species

## Abstract

Aldehyde dehydrogenase 1 (ALDH1) is a cytosolic marker of cancer stem cells (CSCs), which are a sub-population within heterogeneous tumor cells. CSCs associate with therapy-resistance, self-renewal, malignancy, tumor-relapse, and reduced patient-survival window. ALDH1-mediated aldehyde scavenging helps CSCs to survive a higher level of oxidative stress than regular cancer cells. Cruciferous vegetable-derived phenethyl isothiocyanate (PEITC) selectively induces reactive oxygen species (ROS), leading to apoptosis of cancer cells, but not healthy cells. However, this pro-oxidant role of PEITC in CSCs is poorly understood and is investigated here. In a HeLa CSCs model (hCSCs), the sphere-culture and tumorsphere assay showed significantly enriched ALDH^hi^ CSCs from HeLa parental cells (*p* < 0.05). Aldefluor assay and cell proliferation assay revealed that PEITC treatments resulted in a reduced number of ALDH^hi^ hCSCs in a concentration-dependent manner (*p* < 0.05). In the ROS assay, PEITC promoted oxidative stress in hCSCs (*p* ≤ 0.001). Using immunoblotting and flow cytometry techniques, we reported that PEITC suppressed the cancer-associated transcription factor (Sp1) and a downstream multidrug resistance protein (P-glycoprotein) (both, *p* < 0.05). Furthermore, PEITC-treatment of hCSCs, prior to xenotransplantation in mice, lowered the in vivo tumor-initiating potential of hCSCs. In summary, PEITC treatment suppressed the proliferation of ALDH1 expressing cancer stem cells as well as key factors that are involved with drug-resistance, while promoting oxidative stress and apoptosis in hCSCs.

## 1. Introduction

The heterogeneity of the cell population in solid tumors frequently precludes the complete elimination of malignancy after conventional treatments and it can contribute to cancer recurrence. Cancer stem cells (CSCs), which are a subset of tumor cells (0.1–30%) with self-renewal and differentiation potential, are associated with tumor initiation, maintenance, metastasis, and recurrence of a number of cancer types [1,2]. CSCs can survive chemo- or radiation-therapy and they are typically characterized by the expression of drug efflux transporters and anti-apoptotic proteins, enhanced activity of repair enzymes, hypoxic stability, vascular niche, and dormancy [2]. To identify and isolate CSCs from within a heterogeneous population of tumor cells, putative cell surface markers have been proposed, such as CD133^+/hi^, CD24^−/low^, and CD44^+/hi^. While these surface markers are common across various tumor types [3], they are also common to non-malignant embryonic or adult stem cells [4]. More recently, a cytosolic enzyme, aldehyde dehydrogenase family 1, member A1 (ALDH1), has emerged as a functional, intracellular CSCs marker that is associated with malignancy and self-renewal properties of stem cells in many tumors, including breast [5,6], head and neck [7], melanoma [8], hepatic [9], pancreatic [10], colon [11], lung [12], prostrate [13], bladder [14], ovarian [15], endometrial [16], and cervical [17,18] cancers. In addition, high ALDH1 expression showed a strong association with therapy resistance [19].

CSCs-targeted therapy is of growing interest, as conventional chemo- or radiation-therapy does not eradicate drug-resistant CSCs [2]. Activity of drug efflux transporter proteins or ATP binding cassette (ABC) transporters, such as P-glycoprotein (P-Gp) and multidrug resistance (MDR)-associated proteins (MRP) contribute to drug-resistance in CSCs [20]. Particularly, P-Gp (also known as ABC subfamily B1 or ABCB1) having a broad substrate specificity is a major player in therapy resistant cancers [21]. The P-Gp activity is mediated through the expression of multidrug resistance protein 1 (MDR1) gene, whose promoter is activated upon binding with the specificity protein 1 (Sp1) transcription factor [22]. Sp1 is a ubiquitous transcription factor that is involved in cell proliferation and cancer development [23].

The ability of CSCs to survive a significantly higher oxidative stress in comparison to other types of cancer cells or healthy cells also contribute to therapy resistance. Rapidly growing cancer cells produce higher levels of reactive oxygen species (ROS) than normal healthy cells due to increased oxidative metabolism and the faster depletion of nutrient supply [24]. Many cancer therapies exploit this property to preferentially induce apoptosis in tumor cells by increasing the ROS levels further beyond the threshold that a cell can tolerate [25]. The CSCs tend to overcome this higher cancer-therapy induced ROS levels through the anti-oxidation pathways that are associated with glutathione (GSH) and lipid peroxidation enzymes that include the ALDH family of enzymes [26]. ALDH enzymes irreversibly catalyze the oxidation of aldehydes to their respective carboxylic acids, thereby reducing the cellular oxidative stress [27]. Naturally occurring pro-oxidant compounds in cancer patients’ diet may help to complement the effectiveness of cancer-therapies that rely on elevation of ROS as a mechanism of treatment. Working in synergy, they can potentially induce apoptosis in a greater number of tumor cells, including CSCs, leading to a more effective eradication of cancer.

Phenethyl isothiocyanate (PEITC) is a biologically active dietary compound that is derived from cruciferous vegetables, such as watercress, broccoli, cabbage, and cauliflower [28,29]. We and others have reported anti-inflammatory and chemo-preventive characteristics of PEITC [29,30,31,32,33,34]. More recently, we showed that PEITC targets CSCs by promoting apoptosis [34]. Furthermore, PEITC has been shown to deplete GSH and selectively induce ROS-accumulation inside cancer cells (not specifically CSCs), but not in healthy cells [35,36]. However, the pro-oxidant effect of PEITC in CSCs remains poorly understood. HeLa is one of the two cervical cancer cell lines forming spheres among four tested cell lines (i.e., HeLa, CaSki, SiHa, and C33A) and it possesses more cancer stem cell-like properties, such as ability to differentiate, resistance to chemotherapeutics, and CD44^High^/CD24^Low^ CSCs markers, when compared to the other SiHa sphere cells [37]. Since we have already optimized and reported HeLa sphere culture cells in our previous study [34], here we further investigated the CSCs-specific pro-oxidant potential of PEITC using HeLa cervical cancer stem-like sphere culture model (hCSCs) in vitro, followed by a pilot-scale animal study in a non-obese diabetic/severe combined immune deficit (NOD/SCID) mouse model.

## 2. Results

### 2.1. Enrichment of Aldehyde Dehydrogenase^hi^CD44^hi^ HeLa Cancer Stem Cells

**Figure 1:**
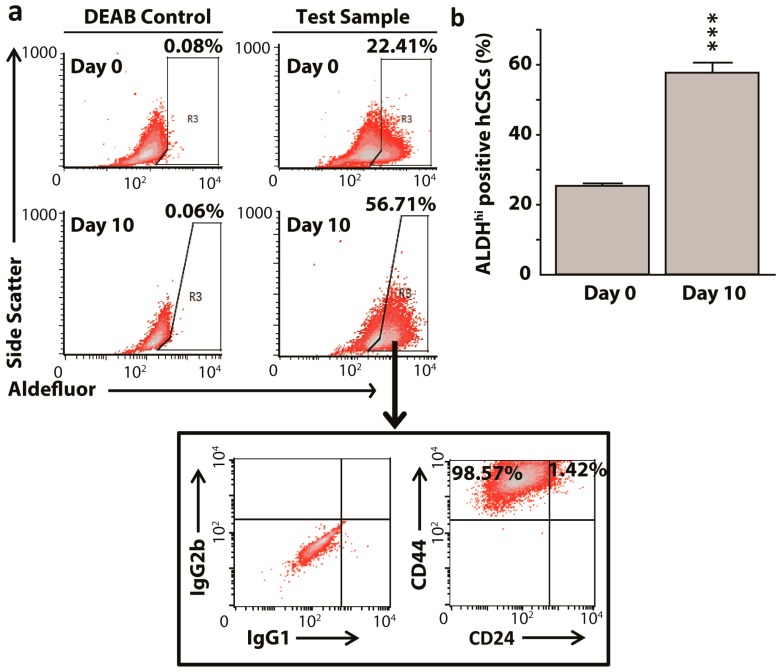
Sphere culture method enriches aldehyde dehydrogenase^hi^CD44^hi^ HeLa cervical cancer stem-like sphere culture model (ALDH^hi^CD44^hi^ hCSCs) population from parental HeLa cells. (**a**) Representative fluorescence-activated cell sorting (FACS) dot plots showing enrichment of ALDH^hi^ cancer stem-like cells from day 0 to day 10 post sphere formation in low anchorage dishes. The ALDH^hi^ gated cells were mostly CD44 positive (**b**). Bar graphs representing aldehyde dehydrogenase enrichment in HeLa cultures showing cancer stem cells enrichment level was significantly higher by end of the tenth day. All data represent means ± SEM, *** *p* ≤ 0.001. DEAB: diethylaminobenzaldehyde.

Others and we have shown that sphere culture enriches the population of sphere-forming CSCs-like cells from the HeLa cervical cancer cell line that can be detected while using cell surface markers [34,37]. Here, we quantified similar enrichment using an intracellular cervical CSCs marker, ALDH1 [18]. Before using the Aldefluor assay for data collection, we optimized the proper concentration of hCSCs and found that 2 × 10^5^ cells/mL produced the optimum signal intensity when compared to 5 × 10^5^ and 1 × 10^6^ cells/mL. After growing the cells in serum free sphere-culture medium, 25.40% of ALDH^hi^ in HeLa cells at day 0 significantly enriched (*p* < 0.001) to 57.75 % at day 10 (Figure 1). The enriched ALDH^hi^ hCSCs corroborated with known cell surface marker characteristics from our previous study, i.e., CD44^hi^ and CD24^low^ cells, thus confirming the sphere-culture mediated enrichment of hCSCs and its detection using ALDH1 (Figure 1). Furthermore, HeLa cells that were grown for 10 days in complete growth medium with 10% fetal bovine serum (FBS) did not show sphere formation or an increase in the proportion of ALDH^hi^ cells.

### 2.2. Phenethyl Isothiocyanate Reduced Aldehyde Dehydrogenase 1 Expressing HeLa Cancer Stem Cells

**Figure 2:**
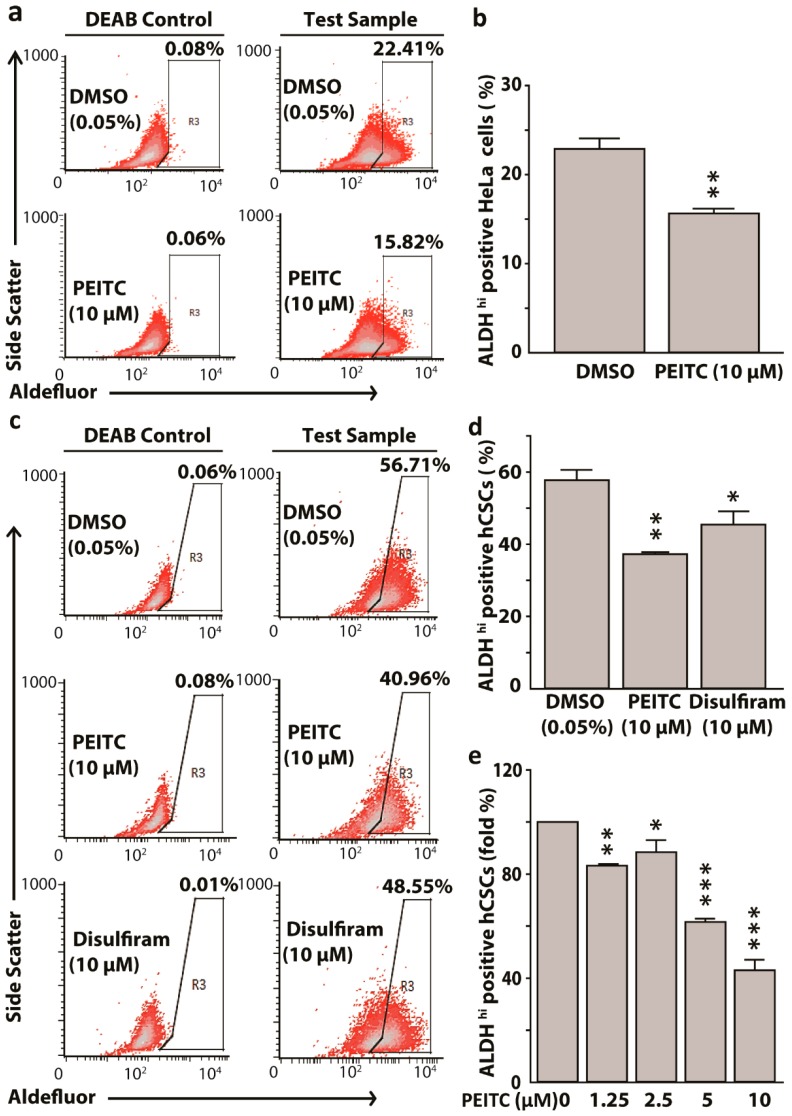
Phenethyl isothiocyanate (PEITC) attenuates aldehyde dehydrogenase 1(ALDH) expressing HeLa cancer stem cells (hCSCs) in a concentration dependent manner. Representative fluorescence-activated cell sorting (FACS) dot plots showing PEITC reduced ALDH1 expressing HeLa cells (**a**) and hCSCs (**c**). Bar graphs showing the reduction of ALDH high cells in HeLa (**b**) and in hCSCs, hCSCs + PEITC, and hCSCs + Disulfiram treatments, using Disulfiram as a known ALDH-inhibiting agent (positive control) (**d**). Bar diagrams showing attenuation of ALDH high hCSCs by PEITC in a concentration dependent manner (**e**). All data represent means ± SEM, * *p* ≤ 0.05, ** *p* ≤ 0.01, *** *p ≤* 0.001. DEAB: diethylaminobenzaldehyde. DMSO: dimethyl sulfoxide control.

PEITC inhibits ALDH2 in the liver [38]. Since NCBI blast revealed that human ALDH1 and ALDH2 share 68% of amino acid sequences, we hypothesized that PEITC can potentially target CSCs with the high expression of ALDH1. PEITC (10 μM) attenuated ALDH^hi^ HeLa cells when compared to dimethyl sulfoxide (DMSO) control (15.82% vs. 22.41%, *p* < 0.01), while using diethylaminobenzaldehyde (DEAB) as a negative control for Aldefluor reagent (Figure 2a,b). PEITC also attenuated ALDH1 enrichment in hCSCs when compared to the DMSO control (40.96% vs. 56.71%, *p* < 0.01), using disulfiram as a positive control (Figure 2c,d) for PEITC treatment. We observed that both PEITC and disulfiram had similar inhibitory effects on ALDH1 expressing hCSCs. Further, we assessed the concentration-dependent effects of PEITC (1.25–10 μM) on ALDH1 reduction in hCSCs. Exposure to 1.25 μM PEITC reduced ALDH^hi^ cells by 20% (*p* < 0.01), whereas 5 and 10 μM PEITC reduced ALDH^hi^ cells by around 40% and 65% (*p* < 0.001), respectively (Figure 2e). Taken together, PEITC treatments resulted in the attenuation of ALDH1^hi^ hCSCs in a concentration dependent manner.

### 2.3. Reactive Oxygen Species Levels Increased in HeLa Cancer Stem Cells after Phenethyl Isothiocyanate Treatments

**Figure 3:**
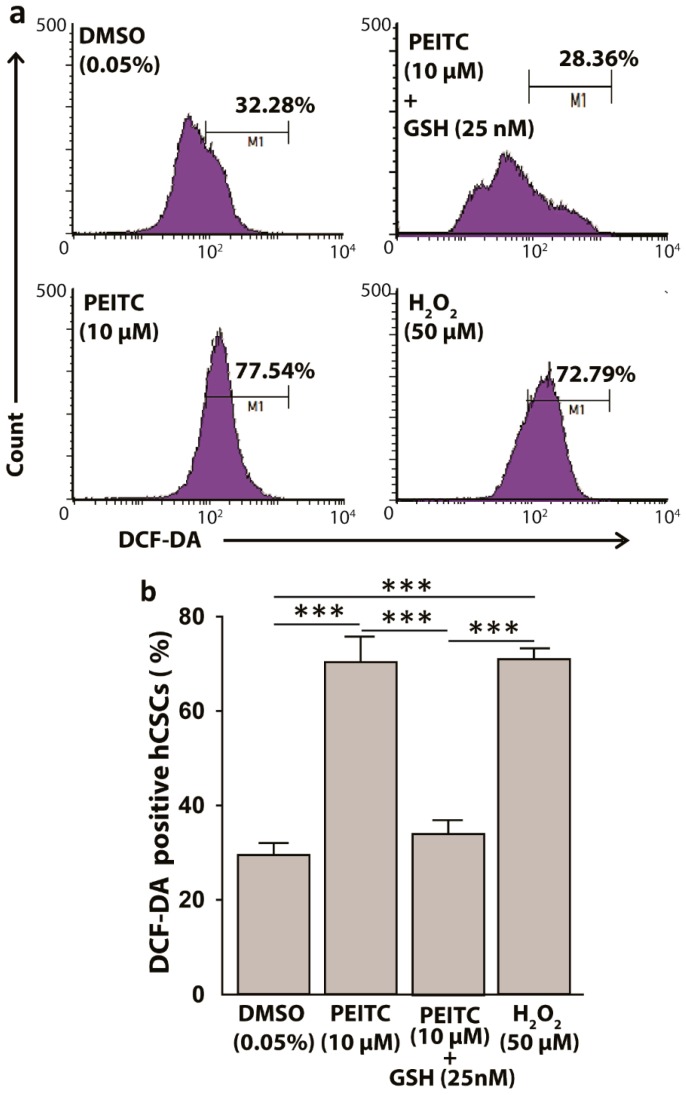
Phenethyl isothiocyanate (PEITC) induces reactive oxygen species (ROS) in HeLa cancer stem cells (hCSCs). (**a**) Representative fluorescence-activated cell sorting (FACS) histograms showing PEITC induces ROS in hCSCs in 3 hr, which can be replenished by exogenous glutathione (GSH). H_2_O_2_ was used as a positive control (**b**). Bar diagram showing the ROS induction by PEITC in hCSCs. All data represent means ± SEM. One-way ANOVA, followed by a Dunnett’s post hoc test, was used to compare multiple means. The pairs with significant mean differences are shown, *** *p* ≤ 0.001. DCF-DA: dichlorofluorescin diacetate.

The electrophilic property of PEITC has been shown to covalently interact with nucleophilic glutathione (GSH), leading to ROS-induction in cells [29]. Since CSCs possess high level of GSH as defensive machinery, we hypothesized a surge in ROS in hCSCs after PEITC treatments. In 2′,7′–dichlorofluorescin diacetate (DCFDA) ROS assay, a 3 h incubation of hCSCs with PEITC (10 µM) as compared to DMSO control significantly increased ROS production by 1.4-fold (*p* < 0.001), which was comparable to the ROS levels that were induced by 50 µM of hydrogen peroxide (H_2_O_2_), a prooxidant positive control (Figure 3). Furthermore, when the PEITC-treated cells were replenished with GSH (25 nM), the prior redox status of the cells were significantly reduced (*p* < 0.001) (Figure 3), supporting the ROS induction by PEITC in hCSCs.

### 2.4. Phenethyl Isothiocyanate Treatment Suppressed HeLa Cancer Stem Cells Proliferation and Increased Early Apoptosis

**Figure 4:**
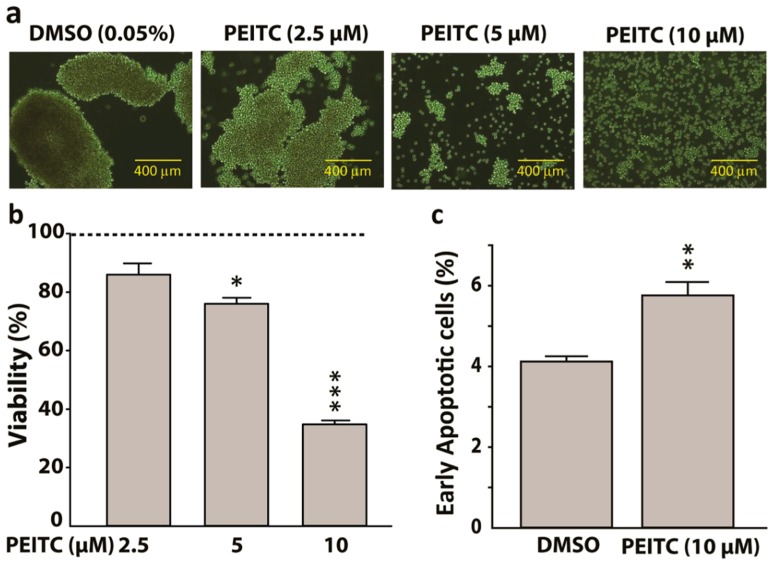
Phenethyl isothiocyanate (PEITC) suppresses HeLa cancer stem cells (hCSCs) proliferation. (**a**) Representative photomicrographs showing attenuation of hCSCs-sphere formation (day 7) by PEITC in a concentration dependent manner (400-μm scale). (**b**) Bar graphs showing concentration-dependent effects of PEITC on the viability of hCSCs after 24 h treatment. The dotted line represents the baseline cell viability for dimethyl sulfoxide (DMSO), which served as a vehicle control to determine statistical significance. (**c**) Bar graphs showing the percentage of early apoptotic cells obtained from Propidium Iodide and Annexin V assay. All data represent means ± SEM, * *p* ≤ 0.05, ** *p* ≤ 0.01, *** *p* ≤ 0.001.

In sphere formation assay, the sphere-forming capacity of hCSCs was reduced by PEITC in a concentration-dependent manner (Figure 4a). In MTS cytotoxicity assay, PEITC significantly lowered the viability of hCSCs by around 20% and 70% when treated for 24 h with a concentration of 5 μM (*p* < 0.05) and 10 μM (*p* < 0.001), respectively (Figure 4b). A common and specific feature of apoptosis initiation or early apoptosis is the transfer of phosphatidylserine from the cytoplasmic surface of the membranes to the cell surface. This externalization contributes to the recognition and subsequent removal of apoptotic bodies by phagocytes and it also provides a binding site for the anionic lipid binding protein annexin V [39]. We showed that PEITC (10 µM) significantly increased the number of Annexin V-positive apoptotic cells (*p* < 0.01). Together, these observations indicate the pro-apoptotic and anti-proliferative properties of PEITC in hCSCs (Figure 4c).

### 2.5. Phenethyl Isothiocyanate Suppressed the Expression of Sp1 and P-Gp Proteins in HeLa Cancer Stem Cells

**Figure 5:**
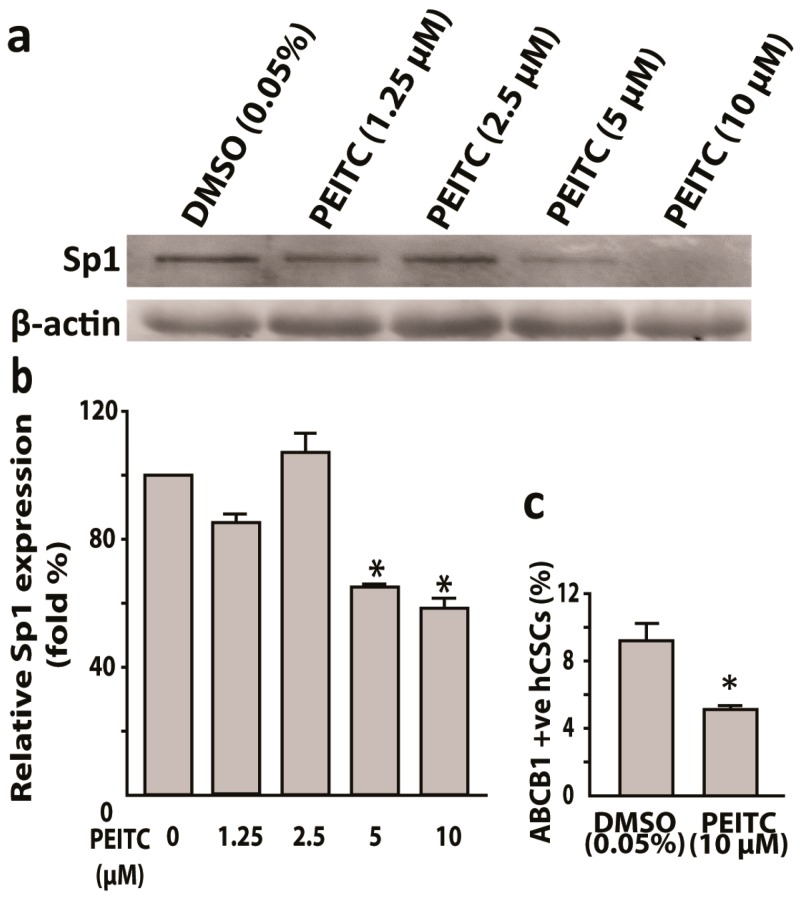
Phenethyl isothiocyanate (PEITC) suppresses transcription factor Sp1 and multidrug resistance protein P-Gp/ABCB1 in HeLa cancer stem cells. (**a**) Western blots showing PEITC attenuated Sp1 and the corresponding bar graphs with relative Sp1 expression (**b**). (**c**) Bar graphs showing 10 µM PEITC suppresses multi-drug resistance protein P-Gp (ABCB1) as compared to vehicle control. All data represent means ± SEM, * *p* ≤ 0.05.

In our previous study [34], hCSCs induced a higher efflux of Hoechst 33342 dye when compared to parental HeLa cells. Hoechst 33342 dye is a substrate of multi-drug transporter P-Gp protein [40]. Thus, we hypothesized PEITC induced the inhibition of P-Gp protein in hCSCs. In flow cytometric analyses, PEITC (10 μM) exposure reduced P-Gp positive hCSCs when compared to DMSO control (4.5% vs. 9%) (Figure 5c). Sp1 transcription factor has been shown to bind to the promoter region of MDR1 (P-Gp) gene [22]. Therefore, as a next step, we examined whether PEITC could inhibit Sp1 protein. Western blot, followed by mean fluorescence intensity analyses, showed that PEITC suppressed the Sp1 protein.

### 2.6. Phenethyl Isothiocyanate Reduced Tumor Initiating Potential of HeLa Cancer Stem Cells

The higher tumor grafting potential of hCSCs and its attenuation by PEITC pretreatment of hCSCs was further evaluated in a small-scale pilot study using a xenotransplantation NOD-SCID mouse model with two test groups (hCSCs/untreated control and 10 µM PEITC-treated hCSCs). Previously, we reported in the same model a higher tumorigenicity of hCSCs when compared to parental HeLa cells [34]. Consistent body-weight and activity among mice from both of the groups were observed throughout the study, indicating that tumor development was not overbearing. An equal number of hCSCs (1 × 10^6^) with 10 μM PEITC pre-treatment (10 µM PEITC-treated hCSCs) developed lower tumor load when compared to untreated control group (hCSCs) at weeks 2 and 4 (Table 1). Furthermore, when compared to hCSCs alone, PEITC-treated hCSCs yielded a lower average tumor volume (0.13 mm^3^ vs. 0.01 mm^3^ and 6.04 mm^3^ vs. 4.3 mm^3^) at week 2 and 4, respectively.

## 3. Discussion

In the absence of rigorous screening programs in developing countries, cervical cancer remains the second-most-fatal cancer in women worldwide [41]. We and others have previously shown that PEITC promotes the death receptor-mediated extrinsic apoptotic pathway in human cervical cancer stem like cells [34,42]. Here, we show that PEITC suppressed the cytosolic functional marker‒ALDH1 expressing hCSCs and the Sp1 transcription factor along with its downstream target P-Gp protein, while promoting oxidative stress and early apoptosis in hCSCs.

Two decades ago, Lindros et al. described a role for PEITC as an inhibitor of mitochondrial ALDH in the liver in the context of alcohol consumption, with similar effects to that of a known ALDH inhibitor, disulfiram [38]. Interestingly, this old concept did not receive much attention until much later, when ALDH1 was proposed as a novel CSC marker for breast cancer [6]. Since ALDH enzymes functionally associate with redox balance in CSCs, we show that PEITC treatments increased the oxidative stress in ALDH1 expressing CSCs and attenuated their proliferation. Although, the early apoptotic cell numbers in response to PEITC exposure are relatively lower than the reduction in cell viability, which is a combined reflection of both early and late apoptotic cells, both data are statistically significant and they support the overall reduced trend of cell proliferation. Furthermore, the apoptosis observed here in PEITC-treated hCSCs is consistent with a relevant study [43], as well as with our previous observation that PEITC exposure upregulates cPARP (apoptosis-associated cleaved poly [ADP-ribose] polymerase), a marker of apoptosis, through DR4 and DR5 (death receptor 4 and 5) of TNF-related apoptosis-inducing ligand (TRAIL) signaling [34].

It is important to note that this study did not aim to test the efficacy of PEITC on diminishing the tumor burden in mice, which would require tumor transplantation, followed by an oral or parenteral PEITC treatment. The animal study aimed to support in vitro observations showing the suppression of hCSCs upon PEITC-treatments. Thus, it was hypothesized that PEITC-pretreatment prior to xenotransplantation in mice would lower cancer initiating or tumor grafting potential of hCSCs that we observed. Our observation is consistent with a prior study that showed in vitro pretreatment of human breast cancer HMLER-shEcad cells with salinomycin, not paclitaxel, resulted in an inhibition of HMLER-shEcad-induced tumorsphere formation [44]. Similarly, our previous study [34] also reported a marked reduction in tumorigenicity and metastasis in mice that had received a PEITC-treated hCSCs. These observations warrant a large-scale efficacy study in the future, similar to that reported by other colleagues [45,46]. Altogether, the animal study helped to further support our in vitro data on effects of PEITC targeting ALDH1^hi^ hCSCs tumorsphere formation.

Although clinical studies are needed to verify the proposed role of PEITC in targeting CSCs that are associated with a range of cancer types, our observation of PEITC attenuating hCSCs-spheres in the context of cervical cancer resonate with a previous report regarding the inhibitory effects of PEITC in colorectal CSCs spheroids [47]. Additionally, PEITC-associated sensitization of chemotherapies through the modulation of P-Gp reported in a cisplatin-resistant gastric cancer cell line aligns with our observations that are presented here [48,49]. In addition, the activation of the Sp1 transcription factor was observed in colon CSCs [22,50] and it was shown as a target for acetylation by histone deacetylase (HDAC) inhibitors [23]. While we reported a new role of PEITC modulating Sp 1 in a stem-like cell population here, PEITC has been shown as an HDAC inhibitor in prostate cancer cells in the context of other genes [43,51]. Therefore, it is possible that an epigenetic regulation is involved in Sp1 suppression by PEITC in hCSCs, which merits future investigation to establish a direct interaction showing the underlying mechanism of PEITC-associated attenuation of hCSCs. Finally, the concentrations of PEITC that were used in this study are achievable following oral administration in humans [52] and they have been used in our previous studies to induce apoptosis in colon cancer cells [32] and cervical cancer stem-like cells [34]. Therefore, including the dietary phytochemical PEITC in the treatment plan for cancer patients and in the maintenance regimens for cancer survivors may help to overcome multi-drug resistance to potentially alleviate the burden of tumor recurrence.

## 4. Materials and Methods

### 4.1. Sphere Cultures of HeLa Cancer Stem Cells

Following our previous work [34], human cervical HeLa cell line (CCL-2, American Type Culture Collection, Manassas, VA, USA) was cultured and maintained in a T-25 flask with Dulbecco’s modified eagle’s medium (DMEM), supplemented with 4 mM L-glutamine, 4.5 g/L glucose (HyClone, Logan, UT, USA), 10% heat-inactivated FBS (Invitrogen, Grand Island, NY, USA), and 1% penicillin (25 U/mL)/streptomycin (25 μg/mL) (Sigma-Aldrich, St. Louis, MO, USA) in a 5% CO_2_-humidified atmosphere at 37 °C. HeLa cells were trypsinized with TrypLE (Invitrogen, Grand Island, NY, USA) and then sub-cultured (1:5 splitting ratio) when the cells were 80–90% confluent. From the parental HeLa cells, CSCs (hCSCs) were cultured following a modified protocol that was described by Gu et al. [34,37]. Briefly, single-cell suspensions of HeLa cells (4 × 10^4^) were seeded into a 100-mm ultra-low attachment (ULA) petri dish (Corning Inc., Corning, NY, USA) containing 8 mL of serum-free mammary epithelial basal medium (MEBM, Lonza, Allendale, NJ, USA), supplemented with 20 ng/mL hEGF, 20 ng/mL hFGF (Invitrogen, Grand Island, NY, USA), 1x B27 (Invitrogen, Grand Island, NY, USA), and 4 μg/mL heparin (Sigma-Aldrich, St. Louis, MO, USA). After an initial five-day culture in suspension at 37 °C, an additional 9 mL of sphere culture medium was added for another five days of culture. On day 10, the spheres were harvested, centrifuged at 500× *g* for 3 min, washed with phosphate-buffered saline (PBS), trypsinized with TrypLE for 10 min at 37 °C, centrifuged at 500× *g* for 3 min, resuspended in 5 mL of hCSCs culture medium, and counted with a hemocytometer. The hCSCs were used for flow cytometry and cell proliferation experiments.

### 4.2. Aldefluor Assay

The Aldefluor Kit (STEMCELL Technologies, Vancouver, Canada) was used to determine the levels of ALDH1 in hCSCs. 24 h prior to cell harvesting, hCSCs were treated with PEITC (Sigma-Aldrich, St. Louis, MO, USA) or DMSO (Sigma-Aldrich, St. Louis, MO, USA). The cells were trypsinized, washed with 1× PBS, and counted using a hemocytometer. Cells were then resuspended in Aldefluor buffer at a concentration of 2 × 10^5^ cells/mL. Activated Aldefluor reagent (300 μM) was prepared following the manufacturer’s instructions. PEITC-treated cells were incubated with activated Aldefluor reagent and its control reagent, diethylaminobenzaldehyde (DEAB) at 37 °C for 45 min, with intermittent shaking after every 15 min. Following incubation, all of the tubes were centrifuged at 250× *g* for 5 min at 4 °C. Aldefluor-stained cells were washed once in Aldefluor buffer and maintained in 4 °C until acquired through FL1 channel in flow cytometer (FACSCalibur, Becton, Dickinson, and Company, San Jose, CA, USA) as ALDH high (ALDH^hi^) and side scatter low phenotypes.

### 4.3. Reactive Oxygen Species (ROS) Assay

The cells were harvested, trypsinized, washed with 1× PBS, and counted using a hemocytometer before collecting in conical test tube. Using Cellular ROS Detection Assay Kit (Abcam, San Francisco, CA, USA), the cells were stained with 20 μM 2′,7′-dichlorofluorescin diacetate (DCFDA) in 1 × Buffer supplied for 30 min at 37 °C following the manufacturer’s protocol. Individual batches of 2 × 10^5^ hCSCs per sample were aliquoted into a six-well plate and then incubated with 10 μM PEITC or DMSO control for 3 h at 37 °C. Glutathione (GSH, Sigma-Aldrich, St. Louis, MO, USA) was used as an inhibitor of ROS production and a pro-oxidant hydrogen peroxide (H_2_O_2_) was used as a positive control in the assay. The read signal was detected at Ex485 nm/Em535 nm using a flow cytometer.

### 4.4. Flow Cytometry

Following our previous study [34], the cells were washed with 2 mL of PBS, trypsinized with 1 mL of TrypLE, and resuspended in 1 mL of PBS, followed by immunostaining. Similarly, hCSCs were collected after 10 days of culture, trypsinized, and resuspended in 2 mL of PBS with a density of 1 × 10^6^ cells/mL, followed by immunostaining. The cells were immunostained with anti-CD24–FITC (1:500 *v*/*v*, Millipore, Billerica, MA, USA) or anti-CD44–FITC (1:500 *v*/*v*, Millipore, Billerica, MA, USA) antibodies for 1 h at room temperature. Immunofluorescence was measured using FACSCalibur cell analyzer (Becton Dickinson, San Jose, CA, USA), with approximately 10,000 events in each sample. Propidium iodide/annexin V staining was performed according to the manufacturer’s instructions. Briefly, the 5 × 10^5^ cells were centrifuged and resuspended in 100 μL of 1 × binding buffer (Invitrogen, Grand Island, NY, USA). The cells were treated with 10 μM PEITC or control (DMSO) for a total of 24 h. The cells were then incubated with 5 μL of annexin V–FITC (eBioscience, Inc., San Diego, CA, USA) and 5 μL of propidium iodide (eBioscience, Inc., San Diego, CA, USA) at room temperature for 5 min in the dark before acquiring in a flow cytometer.

### 4.5. Sphere-Formation Assay

The hCSCs were enriched in spheres in serum-free medium, as described previously [34]. Briefly, cells were treated with 2.5, 5, or 10 µM of PEITC or DMSO as vehicle control. After seven days of incubation, photomicrographs of spheres were taken under an inverted phase-contrast microscope (Olympus America Inc., Center Valley, PA, USA).

### 4.6. Cell Proliferation Assay

3-(4,5-Dimethylthiazol-2-yl)-5-(3-carboxymethoxyphenyl)-2-(4-sulfophenyl)-2H-tetrazolium (MTS) assay was used to determine the number of viable cells, which were seeded into 96-well microplates at a density of 2 × 10^4^ cells per well. Cells were cultured in DMEM that was supplemented with 100 U/mL penicillin, 100 µg/mL streptomycin, 5% heat-inactivated FBS, and 50 µM 2mercaptoethanol. hCSCs were treated with three concentrations of PEITC (2.5, 5, and 10 µM). After 24 h of incubation, 20 µL of CellTiter reagent was added directly to the cell-culture wells and incubated for 1 h at 37 °C, followed by cell viability assessment using the CellTiter 96 AQueous One Solution Cell Proliferation Assay kit (Promega, Madison, WI, USA), containing MTS. The manufacturer’s instructions were followed and the treatments were compared with control (DMSO-treated cells) at 490 nm in a BioTek Synergy H4 multimode plate reader (BioTek, Winooski, VT, USA).

### 4.7. Immunoblotting

The hCSCs (1 × 10^6^ per well) were seeded in a six-well plate and incubated overnight at incubator (37 °C, 5% CO_2_). Old culture media were replaced by culture medium containing 1.25, 2.5, 5, or 10-μM concentrations of PEITC for 3 h. Cell harvesting and immunoblotting were carried out as we previously reported [30]. Briefly, the cells were lysed in ice-cold RIPA buffer containing 150 mM NaCl, 50 mM Tris (pH 8.0), 10% glycerol, 1% Nonidet P-40 (NP-40), and 0.4 mM EDTA, followed by a brief vortexing and centrifuging at 14,000× *g* for 15 min at 4 °C. Equal amounts of cell lysate proteins were separated by SDS-PAGE through a 12% separating gel, transferred to nitrocellulose membranes that were blocked with 5% non-fat dry milk, and probed overnight at 4 °C with mouse anti-human Sp1 (1:1000 *v*/*v*, Millipore, Billerica, MA, USA). Rabbit anti-human β-actin (1:5000 *v*/*v*, Millipore, Billerica, MA, USA) antibody was used as a housekeeping protein. Blots were then washed in PBS and further incubated with secondary antibodies, goat anti-mouse horseradish peroxidase (1:3000 *v*/*v*, Millipore, Billerica, MA, USA), and goat anti-rabbit horseradish peroxidase (1:5000 *v*/*v*, Millipore, Billerica, MA, USA), for 1 h at room temperature. Finally, after rinsing in PBS with 0.1% Tween20, the blots were incubated for 5 min in enhanced chemiluminescent substrate for horseradish peroxidase (SuperSignal™ West Femto, Thermo Fisher Scientific, Waltham, MA, USA) and imaged with a ChemiDoc XRS+ Imaging System (BioRad, Hercules, CA, USA), followed by a mean fluorescence intensity analysis.

### 4.8. Mice Tumorigenicity Study

Following the guidelines and approval of the Institutional Animal Care and Use Committee (IACUC), South Dakota State University (IACUC approval #12-087A, approved on 12 December 2012), eight female (five-week old) non-obese diabetic, severe combined immunodeficient (NOD/SCID, NOD.CB17-*Prkdc*^scid^/J) mice (Jackson Laboratories, Bar Harbor, ME, USA) were randomly grouped into two groups (four mice per group) in a specific pathogen-free (SPF) housing at a constant temperature of 24–26 °C, with a 12-h:12-h light/dark cycle. All of the mice were provided with autoclaved standard natural ingredients chow diet (Envigo, Somerset, NJ, USA) and autoclaved water ad libitum, starting from one-week of acclimatization period prior to the treatment. In the meantime, hCSCs were cultured, trypsinized, washed, pre-treated with 10 µM PEITC for 24 h, and resuspended in PBS at the concentration of 1 × 10^7^ cells/mL prior to injection. Each mouse was subcutaneously injected at both flank regions, with one injection of PBS (100 µL, control group), hCSCs (1 × 10^6^), or hCSCs (1 × 10^6^) pretreated with 10 µM PEITC. The cell number in each injection was consistent with the study that was previously carried out by Gu et al. [37] and Wang et al. [34]. All of the mice were routinely monitored for tumor formation, weight loss, pain, and distress. The mice were euthanatized with CO_2_ inhalation 28 days post-treatment and the average tumor number per injection was calculated in each group.

### 4.9. Statistical Analysis

Sigma Plot software (Systat Software, Inc., San Jose, CA, USA) was used for the statistical analyses. Analysis of variance (ANOVA), followed by a Dunnett’s post hoc test, was used to compare multiple means, while statistical significance was assessed by Student’s *t* test when a comparison was made between two groups receiving similar treatments. Data were expressed as means ± SEM. Experiments were repeated at least three times. The level of statistical significance was denoted by asterisks: * *p* ≤ 0.05, ** *p* ≤ 0.01, and *** *p* ≤ 0.001.

## 5. Conclusions

PEITC treatments suppressed the proliferation of hCSCs expressing higher levels of intracellular CSCs marker ALDH1. An increase in oxidative stress and early apoptotic cells in PEITC-treated hCSCs was observed. The in vitro observations of hCSCs-targeting effects of PEITC associated with a lower tumorigenic potential of PEITC-pretreated hCSCs in vivo. In addition, PEITC treatments attenuated the transcription factor Sp1 and its downstream target, a drug efflux transporter protein P-gp, which further supports a potential role for PEITC in targeting therapy-resistant CSCs in the context of cervical cancer. Vegetable derived PEITC may be further investigated for its use as a complementary intervention for the mitigation of cancer relapse and the alleviation of associated healthcare burden.

## Figures and Tables

**Table 1:** Tumor development in non-obese diabetic, severe combined immunodeficient (NOD/SCID) mouse model.

Injection (1 × 10^6^ Cells)	Tumors at Week 2	Tumors at Week 4
hCSCs	1/8	4/8
10 µM PEITC-treated hCSCs	0/8	1/8

hCSCs: HeLa cancer stem cells; PEITC: Phenethyl isothiocyanate; NOD/SCID: Non-obese diabetic/severe combined immunodeficient.

## Abbreviations

ALDH1	Aldehyde dehydrogenase 1
CSCs	Cancer stem cells
DMSO	Dimethyl sulfoxide
hCSCs	HeLa cervical cancer stem cells
MDR	Multi-drug resistance
NOD/SCID	Non-obese diabetic, severe combined immunodeficient
PEITC	Phenethyl isothiocyanate
ROS	Reactive oxygen species
